# Design and Purchase Intention Analysis of Cultural and Creative Goods Based on Deep Learning Neural Networks

**DOI:** 10.1155/2022/3234375

**Published:** 2022-08-29

**Authors:** YuanHong Sun

**Affiliations:** Kookmin University, Seoul 02707, Republic of Korea

## Abstract

With the rise of cultural and creative industries, cultural creativity has gradually become an important factor to promote the value of design in the future, and it is also a trend to integrate “cultural elements” into the design of products. At present, the cultural and creative industries in Western countries and Taiwan are the mainstay of their economic development. We should actively absorb their successful experiences and, with the support of national policies, carry out effective and lasting development of them, as well as continuously improve the quality of cultural and creative products. In today's steady economic development, the emotionalization of cultural and creative consumption has gradually formed a new consumption trend. When cultural and creative consumers buy stationery, they will inevitably have three situations: purchasing instinct, purchasing behavior, and reflection. Therefore, this paper adopts the method based on machine learning to conduct in-depth research on the users of cultural and creative products of “Forbidden Day and Night Set Gift Box.” Through the research on the cultural and creative consumption intention of “Forbidden Day and Night Set Gift Box,” it can effectively promote the development of domestic cultural and creative enterprises, and then promote the customer satisfaction of cultural and creative enterprises. This paper makes a detailed analysis of it from the perspectives of data processing, feature engineering, classification prediction models, and future development directions. By studying the cultural and creative behavior of users, a deep learning model based on neural network is established. The feature extraction, feature preprocessing, feature selection, and asymmetric data collection in the process of data processing are discussed in depth. In order to further improve the prediction accuracy and conduct more in-depth research, this paper establishes a deep learning prediction model based on depth. This model is experimentally validated and it can be observed that the model is 10% more efficient than the traditional model, so the model can learn data better from the user's behavior in several aspects, and the proposed and practice of this model has good practical significance.

## 1. Introduction

As time goes by, people are more and more interested in cultural and creative products with originality and scarcity. Designers are also beginning to turn their attention to graphic design with regional characteristics and to practice regionalized design on this basis. With a history of over 2,500 years, Suzhou has a rich cultural heritage that can be mined for countless patterns that can be analyzed in terms of monuments, architecture, people, customs, and food.

The research objective of cultural and creative consumption behavior is to determine the reference value of creative products and the potential purchasing power of cultural and creative consumers for cultural and creative products [1]. Cultural and creative consumption behavior is an important step to achieving product positioning. In the process of cultural and creative consumption, scholars from different industries have put forward their own views. The analysis of consumers in this paper mainly includes qualitative analysis, quantitative analysis, machine learning models, and recommendation algorithms. Previous research on cultural and creative consumption behavior mainly relies on theoretical methods such as marketing, psychology, and behavior and has explored the marketing behavior of cultural and creative products in depth.

This paper takes the consumption behavior of cultural and creative products of “Forbidden Day and Night Set Gift Box” as the starting point and adopts literature analysis, case analysis, comparative research, and other research methods, in order to find a new way of anticipation and improve the analysis mode. By comparing the most relevant data, this paper believes that the quantitative research on cultural and creative consumption will be more in-depth by establishing an efficient model. Although it only appeared recently, it already has good prospects for development in the fields of social networking, search engines, ad clicks, etc. Deep learning technology can deeply reveal the interrelationships between attributes; therefore, deep learning methods will become an important research field in the future. The article comprehensively sorts out the problem of cultural and creative consumption in the Forbidden Day and Night Set Gift Box from different perspectives. Due to the huge amount of information of cultural and creative users and the tedious processing, the currently available models cannot solve the above problems. In order to improve the effectiveness of prediction, some scholars apply machine learning models and recommendation mechanisms to specific domains. The deep learning technology deeply explores the user characteristics, which will surely have a new impact on the research of cultural and creative consumption in China.

## 2. Deep Learning Model for Consumer Behavior Analysis and Prediction

### 2.1. Cultural Products

**Figure 1:**
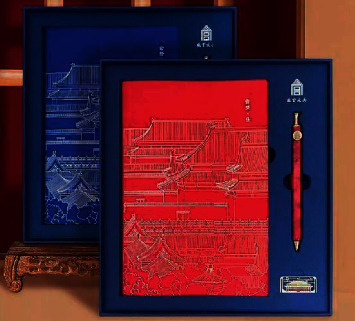
“Forbidden day and night set gift box” cultural and creative products.

Cultural creativity, originating from the cross-integration of cultural creativity, is an abstract concept. When people endow the product with culture and creativity, it gives life and vitality to the cultural and creative industry and gives the product a deeper connotation. In the long development process of China and the world, there are countless excellent cultural inheritances and evolutions. They combine with modern art to present infinite possibilities. It seems to have broken through the barriers of time, and ancient culture and modern art are perfectly integrated. Many interesting cultural and creative products are inspired by the bits and pieces of life, and then combined with traditional culture, constitute a unique and beautiful landscape of the contemporary consumer goods market [2–10]. This paper takes the cultural and creative products of “Forbidden Day and Night Set Gift Box” as the object of investigation, and uses deep learning technology to conduct in-depth mining of user characteristics (as shown in Figure 1).

### 2.2. Deep Learning

In 2006, “Science” published a paper by Hinton, a great machine learning master and his disciples, and introduced it into in-depth research, which triggered a frenzy about in-depth research and received a lot of attention in the industry. A very comprehensive summary was made [11]. Since deep learning was proposed decades ago, it has developed rapidly. With the introduction of various models such as deep neural network, convolutional neural network (CNN), recurrent neural network (RNN), long short-term memory neural network (LSTM), they have gradually developed into the first technology in the field of data mining and machine learning. Aiming at the problem that the neural network is easy to fall into the local minimum and the learning ability is limited, using the idea of “layer-by-layer greedy learning,” by learning a deeper nonlinear network, approximating the composite function, more accurate results can be predicted [12]. This paper proposes a DNN-based customer purchasing strategy for cultural and creative products.

Most of the current “deep learning” models are based on neural networks, which can effectively represent complex functions and do not change due to changes in the data itself during the learning process, making them more robust. In big data mining and predictive analysis, deep learning is a very useful method, which can combine the basic features of the surface layer to mine more high-level abstract features.

### 2.3. Deep Neural Networks

**Figure 2:**
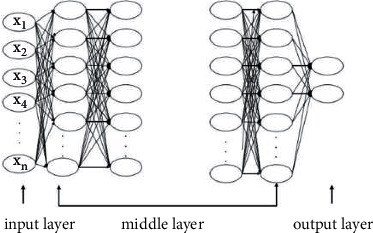
Structure diagram of deep neural network.

Deep neural networks are currently an important research direction in deep learning [13]. Compared with the traditional shallow neural network, the deep neural network adds a new hierarchical structure to the middle layer of the neural network, so that it has a variety of different learning methods, which can be extracted from a large amount of data. It is more effective for classification and prediction characteristics. The number of neurons will increase with the increase of the number of hidden layers, and the increase of the number will inevitably obtain more characteristic information from the data, thereby greatly improving its prediction effect. DN technology has been widely used in many aspects, among which DNN technology can effectively reduce the error rate of speech; in terms of target recognition, the use of DNN technology can improve the recognition accuracy by 10% [14]. On this basis, the learning performance of a deep neural network is better than that of a shallow network, and its architecture is given (the structure diagram is shown in Figure 2).

In the construction of DN, one and two are the input and output, and the others are hidden. It is similar to the traditional neural network, but the difference lies in its training mechanism. In this paper, we propose a hierarchical training method based on hierarchical forward training, whose main purpose is because the fitting speed of neurons at different levels is different, and the training speed is also different. The forward transfer algorithm for deep neural networks is illustrated below (Table 1).

In the context of big data, the DNN model can perform multiple nonlinear fitting methods from the bottom layer to discover the features hidden in the data [15]. Although the DNN model has good feature learning performance, it still has some problems in the analysis and prediction of users' needs: 1) Multiparameter: the structural design of the DNN model is very important for the establishment of the model, so the number of hidden layers must be adjusted, as must neuron number and activation function. (2) Training period: the DNN model has a higher level and has a larger weight, so a larger training period is required.

### 2.4. Building the *r* DNN Model

**Figure 3:**
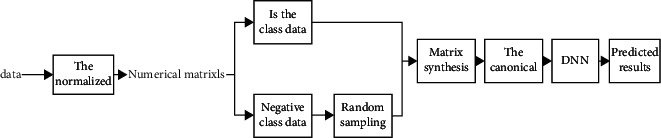
rDNN model structure.

rDNN is an extended mode of DNN, which is different from DNN but adopts a new method to deal with unbalanced data. The model construction of the rDNN is shown in Figure 3:

Construct an improved rDNN model whose purpose is to maintain the DNN model mining depth and reduce the impact of imbalance. The rDNN model can reduce the training cost of the model, reduce the redundancy of negative types of samples, the underlying knowledge learning characteristics, and the more meaningful mining, so as to analyze and predict the behavior of cultural and creative users.

### 2.5. Choosing an Activation Function

**Figure 4:**
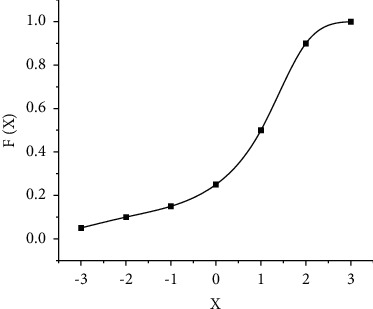
Sigmoid function image function.

**Figure 5:**
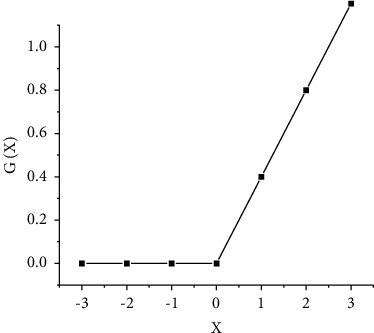
Relu function image.

To overcome the multiple parameters of DNN, this paper determines the number of nodes in each layer by adjusting the layers and activation functions of the neural network [16]. In the DNN mode, the selection of features is particularly important. It is to retain, map, or delete features through functions, and “activation” is the activation of neuron features, so neural networks can handle these problems well. Among them, the most common are Sigmoid, Tanh, Relu, and other incentive functions. The Sigmoid function is also known as the S function. The input value range of this function is (−*∞*, +*∞*), and the output value range is (0, 1), as shown in Figure 4:

Sigmoid is a nonlinear function with a certain probability interpretation function, and Sigmoid is differentiable and easy to be excited at 0, so it is very suitable for neural network modeling [17]. Another common startup function is “Relu,” which is a segmented input value of 0, and if the input value of the input exceeds 0, the input value is entered, as shown in Figure 5.

In recent years, the research and application of Relu has received a lot of attention from scholars at home and abroad. The slope of Sigmoid function is 0 in the positive and negative saturation phase, and the slope of Relu remains constant above 0. Relu has a high operational speed, so the research of Relu in its application is getting more and more attention.

### 2.6. Choose the Appropriate N/P Ratio

**Figure 6:**
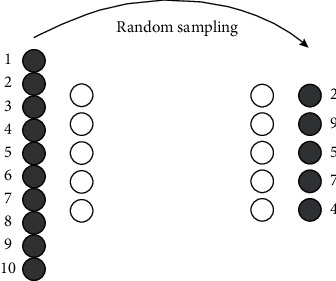
Schematic diagram of N/P.

In order to reduce the impact of data type imbalance on the schema, the ratio of positive and negative types is also called the ratio of negative to positive. For the convenience of labeling, all negative types and ratios of types are set as N/P ratios [18]. In this paper, in the negative category, from normal sampling and negative sampling, the number selected is proportional to the number of normals (see Figure 6).

Through the analysis of the N/P ratio, the appropriate positive and negative samples can be selected, thus overcoming the singularity of the data in a certain sense and improving the universality of modeling. Take black as negative, white as positive, negative as unit, positive as unit, and positive as unit for sampling.

## 3. Model Construction and Algorithm Implementation

### 3.1. System Framework

**Figure 7:**
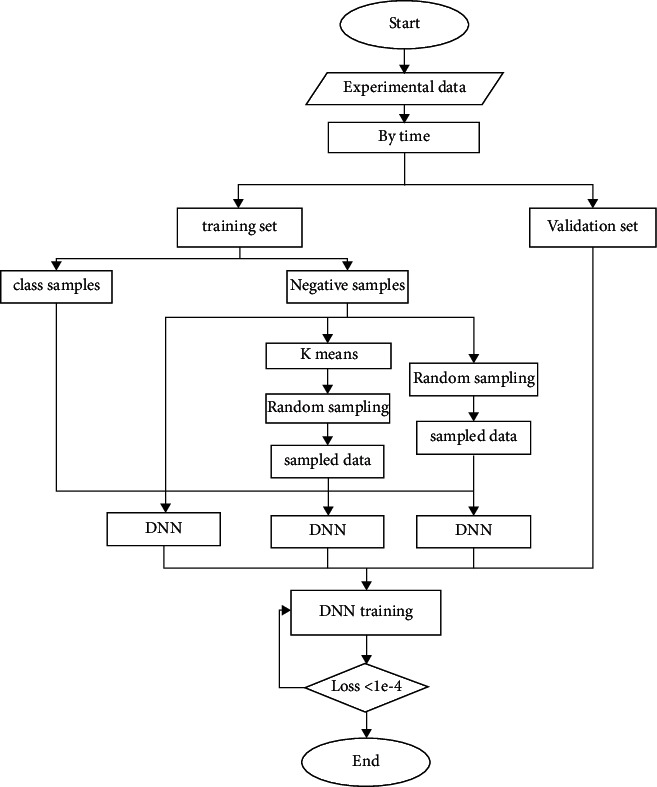
Deep learning model process.

By classifying the samples, a sample-based deep learning model is established (see Figure 7). When constructing a deep network, the DNN, rDNN, and KmDNN modes are first compared and compared, and finally a conclusion is drawn. KmDNN is a new DNN mode based on random sampling of the negative class sample set, which is only used for comparison.

On this basis, rDNN and KmDNN modes are proposed, respectively. On this basis, the early-stop method is used in this case . After increasing the training frequency, the value of loss will not decrease with time. Since this experiment requires a lot of data and has a certain degree of sparseness, plus some artificial data characteristics are appropriately added, plus the limitations of the experimental environment, the whole process of the experiment has no GPU for parallel computing, so the experiment takes more time.

### 3.2. Implementation of the rDNN Model

The method adopts a python-based MiniBatchK Means clustering method, which adopts a random sampling method, and establishes a random model on Python, and generates a random number through the model to randomly sample the model. The development of DNN takes advantage of the modularity and scalability of Keras, and provides more Chinese documents as a reference, which is suitable for beginners to conduct in-depth research. The specific algorithm of rDNN mode is implemented according to the following procedure: (Tables 2–4)

1. Train data performs random sampling after using random numbers generated by the random module to balance the data set:
2. Use Keras to build a DNN network, use SGD's batch gradient descent method, Relu start function, early-stop, in increasing training, the value of loss will not decrease over time until the end of rDNN practice.
3. Adjust the relevant parameters of the model, the comparison of AUC and F.

## 4. Experiment Results and Analysis of Deep Learning Model

### 4.1. Model Parameter Settings

#### 4.1.1. DNN

DNN is a deep neural network that classifies and predicts experimental data and reduces losses by using stochastic gradient descent algorithms. The parameters of DNN are set in Table 5.

#### 4.1.2. rDNN

rDNN is a deep neural network with random sampling based on negative categories. The negative samples in the training data are randomly sampled, and they are sampled with positive category data to form Random_ data, and then the DNN mode is constructed, and its parameters are set. These DNN parameters are set as in Table 5.

#### 4.1.3. KmDNN

KmDNN is a deep neural network based on the sampling of negative category data, which is characterized by training Kmeans clusters of negative categories and randomly selecting negative samples from the clustered groups, and forming KRandom_data with positive category data. Then the DNN mode is established, and its parameters are set as (1) as shown in Table 6.

### 4.2. Experimental Results and Analysis

Due to the use of DNN mode and different sampling methods to conduct experiments, each experiment will produce different results, so more than 30 experiments are carried out, and the average results are used as the final conclusion (Table 7).

As can be seen from Table 7, the improved DNN method is of great help in improving AUC. The rDNN mode is based on the DNN mode, and the prediction efficiency is greatly improved by inverse sampling. Therefore, reducing the positive and negative difference in ratios is very helpful for the prediction of the model because of the large amount of data in this paper, under the conditions of test conditions, test platforms, etc., only random sampling with negative sampling is carried out, and subsequent experiments will compare unbalanced data, such as repeated sampling. In practical applications, the over-sampling method has great defects. There are 7,507 samples and 2,106,772 negative samples. The over-sampling method increases the number of samples and takes up a lot of storage space and computing time which is not ideal. Therefore, the random sampling method can better meet the actual needs. The application of the KmDNN mode of Kmens clustering has also been improved, but when clustering different clusters, the number of clusters needs to be set in advance. Since different algorithms have different compatibility and theoretical interpretability, the different clusters have different negative values, so that random and equal probability cannot be guaranteed during sampling, resulting in data skew. The results show that the neural network model based on DNN has better learning performance and stronger feature expression.

**Figure 8:**
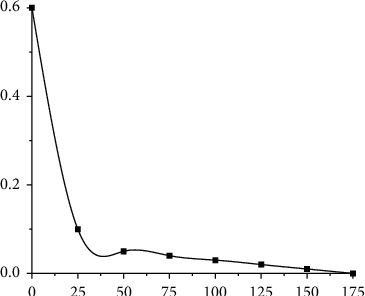
rDNN iterative process.

Through image trials on the training set and validation set of rDNN (Figure 8), after 160 iterations, it decays slowly and trains well. Finally, the DNN-based model was compared with the modified experimental data, and the following points were drawn:

1. The improved KmDNN and rDNN models are better than DNN, reducing the imbalance ratio before establishing the model and improving the prediction efficiency of the model.
2. Comparing DNN with traditional analytical and forecasting models, the results show that deep feature learning can make this model achieve better results.
3. In terms of analyzing and forecasting the needs of users, the rDNN mode is adopted, which not only maintains the depth characteristics of DNNs, but also maintains the high-level abstract characteristics of traditional DNNs, and introduces a processing scheme of unbalanced classification, which reduces the cost of DNNs. workload and improves the operability of the network.

## 5. Conclusion

“Cultural creativity” refers to any product or combination of products produced in the field of “cultural creativity.” It includes the content of cultural creativity and the carriage of hardware. Compared with other products, the core of cultural and creative products is the cultural and creative content. Therefore, its price constitutes the emotional value and connotative spirit of cultural creativity. At the level of consumer behavior, product design is closely related to the feedback link of consumption, and in the daily consumption of consumers, cultural and creative consumers attach great importance to this aspect of activities, which is related to their needs and energy. is fully reflected. Cultural creativity has a direct connection with cultural creativity. Therefore, designers should also pay attention to the experience of the work while carrying out this kind of design. Main the functionality and function of the product. Cultural and creative products should pay attention to the unique principle of creative consumers enjoying consumption. In a sense, this unique experiential design must not only be reflected in function but also grasp the effect of combining with culture. The cultural and creative consumers also trust the effect from product quality. Under the joint action of these three ideas, creative works have the value of cultural exchange.

Firstly, from the traditional cultural and creative consumption behavior models and deep learning models, various models are summarized and summarized through theoretical derivation, model construction, algorithm implementation, and theoretical derivation and practical application. On the basis of DNN, the deep learning model of DNN is analyzed in depth and improved, and the experiments prove the superiority of rDNN in prediction. In this paper, mathematical models such as mathematical regression, random forest and neural network are used and compared with them. For the impact of traditional prediction models on data characteristics, this paper has conducted an in-depth research on them from mathematical significance. Thirdly, by constructing a prediction model for cultural and creative customers, the market share is improved, and thus the company's profit is enhanced. In this paper, we first introduce a deep learning model to improve its prediction accuracy, and based on this, we propose a deep learning-based RDNN model for the classification of unbalanced data. The rDNN model built in this paper enables us to better understand the past consumption habits of consumers of the “No Day No Night” series, find their rules, and maximize the promotion.

## Figures and Tables

**Table 1:** Forward propagation algorithm of deep neural network.

Input: Let the total number of layers be N, the matrix corresponding to all hidden layers and output layers is *w*, the bias vector is *b*, and the input is *X*
Output: a^N^
Initialization: a^l^ = *x*
For *n* = 2 to *N*
*a*^*n*^=*σ*(*z*^*n*^)=*σ*(*w*^*n*^*a*^*n*−1^+*b*^*n*^)
Output: a^*N*^

**Table 2:** Randomly extracted data.

Import random
nNowCount = 0
forRandom_data = open (train_data, “*W*”)
For line in open (strFeatureMatNormal);
If listSerial [nNowCount] = = 1;
Fout Random_data.write (line)
nNowCount+ = 1
Print “Random_data is OK”

**Table 3:** rDNN model training process.

Model.compile (loss = “mean_squared_error,” optimizer = “sgd,” metrics = [“accuracy”]
Early_stopping = EarlyStopping (monitor = “val_loss,” patience = 50)
History = model.fit (data, label, batch_size = 100, nb_epoch = 200, shuffle = True, callbacks = [early_stopping])

**Table 4:** Calculation of evaluation indicators.

From sklearn import metrics
f1 = metrics.f1_source (test_*y*, test_*y*_pred)
auc = metircs.roc_aue_source (test_*y*, predict_prob_*y*)

**Table 5:** DNN parameter settings.

Parameters of the item	Set
Model structure	128-64-64-30-2
The objective function	Binary_crossentropy
Train the maximum number of iterations	200
The activation function	Relu

**Table 6:** *K*_means parameter.

Parameters of the item	Set
Maximum iteration	200
Clustering number	1000
Extract subset size	200

**Table 7:** Comparison of experimental results.

Deep learning model	On average, AUC
DNN	0.7893
R DNN	0.8322
Km DNN	0.8064

## Data Availability

The datasets used in this paper are available from the corresponding author upon request.
